# Metabolic follow-up of a Croatian patient with gyrate atrophy and a new mutation in the *OAT* gene: a case report

**DOI:** 10.11613/BM.2018.030801

**Published:** 2018-10-15

**Authors:** Marija Zekušić, Ana Škaričić, Ksenija Fumić, Dunja Rogić, Tamara Žigman, Danijela Petković Ramadža, Nenad Vukojević, Véronique Rüfenacht, Valentina Uroić, Ivo Barić

**Affiliations:** 1 Department of Laboratory Diagnostics, University Hospital Center Zagreb, Zagreb, Croatia; 2 Department of Pediatrics, University Hospital Center Zagreb, Zagreb, Croatia; 3 School of Medicine, University of Zagreb, Zagreb, Croatia; 4 Department of Ophthalmology, University Hospital Center Zagreb, Zagreb, Croatia; 5 Division of Metabolism, University Children’s Hospital, Zürich, Switzerland; 6 Department of Nutrition and Dietetics, University Hospital Center Zagreb, Zagreb, Croatia

**Keywords:** ornithinemia, amino acids, tandem mass spectrometry

## Abstract

Gyrate atrophy (GA) of the choroid and retina is a rare autosomal recessive disorder that occurs due to deficiency of the mitochondrial enzyme ornithine aminotransferase (OAT). Hyperornithinemia causes degeneration of the retina with symptoms like myopia, reduced night vision and progressive vision loss. Our patient is a 10-year-old girl with impaired vision and strabismus. As part of the metabolic work-up, plasma amino acid analysis revealed significantly increased concentration of ornithine (1039 μmol/L; reference interval 20 - 155 μmol/L). Molecular genetic analysis revealed homozygous mutation in exon 7 of the *OAT* gene that has not been reported previously (c.868_870delCTT p.(Leu290del)). This in frame deletion was predicted to be deleterious by *in silico* software analysis. Our patient was treated with pyridoxine (vitamin B_6_ in a dose of 2 x 100 mg/day), low-protein diet (0.6 g/kg/day) and L-lysine supplementation which resulted in a significant reduction in plasma ornithine concentrations to 53% of the initial concentration and the ophthalmologic findings showed significant improvement. We conclude that low protein diet and lysine supplementation can lead to long-term reduction in plasma ornithine concentrations and, if started at an early age, notably slow the progression of retinal function loss in patients with GA. The effect of therapy can be reliably monitored by periodical measurement of plasma ornithine concentration. To our knowledge, this is the first report of OAT deficiency in Croatia.

## Introduction

Gyrate atrophy (GA) of the choroid and retina (OMIM: 258870) is an inherited disorder of ornithine metabolism characterized by slowly progressive vision loss (*1*). This condition is caused by mutations in the ornithine aminotransferase (*OAT*) gene and is inherited in an autosomal recessive manner, resulting in significantly reduced activity of mitochondrial enzyme ornithine aminotransferase (OAT; EC 2.6.1.13) (*2*). As a consequence, patients have markedly elevated concentrations of ornithine (10 to 15 times higher than normal) in plasma and other body fluids (*3*). Ornithine aminotransferase utilizes pyridoxal 5-phosphate, an active form of vitamin B_6_ as a co-factor and is expressed in most tissues, including kidney, small intestine, liver, and retina (*4*).

The *OAT* gene analysis is helpful for diagnosing patients with suspected GA. This gene encodes the mitochondrial enzyme OAT which is a key enzyme in the pathway that converts arginine (Arg) and ornithine (Orn) into glutamate (Glu) (*5*). The OAT protein is coded by a single gene, located on chromosome 10, region q26 (*6*). The primary transcript of the *OAT* gene is 21 kb long and contains 11 exons (*7*). Translation starts in exon 3 and seems to occur on free polysomes (*8*). To date, 68 pathogenic mutations have been identified on ClinVar’s website, plus 36 likely pathogenic, 25 of uncertain significance, 3 likely benign and 4 benign mutations (*9*).

The clinical manifestation of GA usually starts with deterioration in visual acuity and night vision, followed by the appearance of sharply demarcated, circular areas of chorioretinal atrophy with hyperpigmented margins in the midperiphery of the fundus (*10*). During the second and third decade of life the areas of atrophy enlarge and the ocular damage becomes irreversible. By the end of the second decade, most patients with GA develop cataract (*11*). These progressive vision changes usually lead to blindness in the fifth or sixth decade of life. The exact mechanism of chorioretinal degeneration remains unknown but it is believed to be due to a toxic effect of ornithine or one of its metabolites. Gyrate atrophy is a genetic disorder with increased incidence in Finland, estimated at 1:50,000 (*10*). Treatment options include dietary supplements and/or a specialized diet. The main source of Orn is dietary Arg, and restriction of Arg in the diet appears to have a therapeutic effect (*12*).

The aim of this case report is to present the follow-up of treatment of a 10-year-old girl with GA and points out the importance of regular measuring of amino acid concentrations during the patient’s follow-up.

## Materials and methods

### Subject

**Figure 1:**
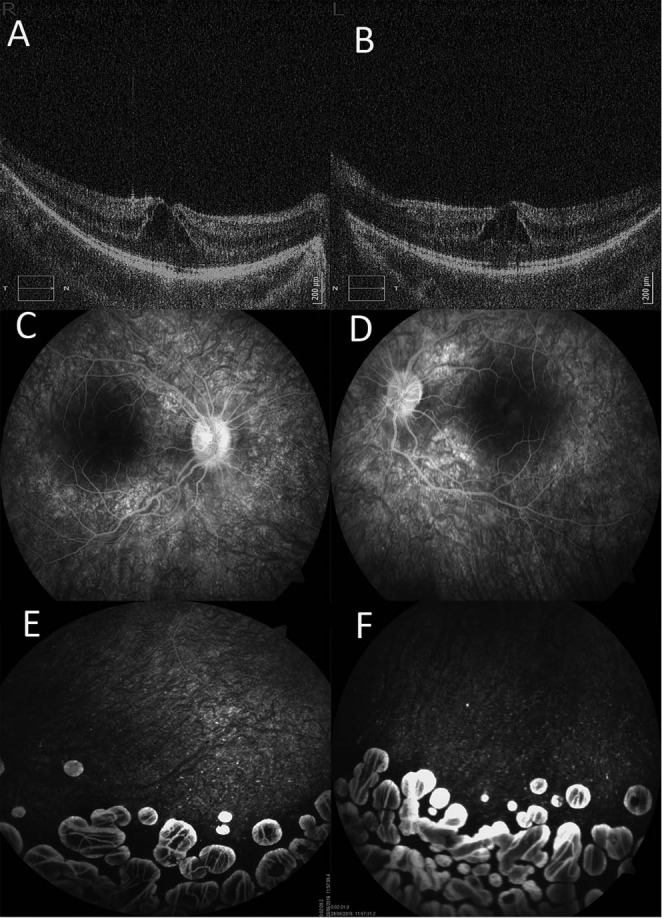
Bilateral cystoid macular oedema - optical coherence tomography (A,B), fluorescein angiography (C,D) and peripheral fundus with numerous circular sharply limited atrophic zones in the retina - fluorescein angiography (E,F).

Here we present a case of a 10-year-old girl with GA and bilateral cystoid macular oedema. First symptoms in our patient appeared at the age of four when she experienced partial vision loss and strabismus in General Hospital Pula, Croatia. No other family member has reported any eye problems and her parents were both healthy and unrelated. At the age of six years, large retinal atrophic area was observed and GA of the choroid and retina was suspected. Because of this characteristic ophthalmologic finding, she was further evaluated in the University Hospital Centre Zagreb, Croatia under suspicion of OAT deficiency. Ophthalmologic findings, optical coherence tomography (Copernicus, Optopol Technologies, Zawierci, Poland) and fluorescein angiography (Visucam 500, Carl Zeiss Meditec, Jena, Germany) revealed bilateral macular oedema, with numerous circular sharply limited atrophic zones in the retina (Figure 1). Therapy with systemic corticosteroids and topical carbonic anhydrase inhibitors was administered. Further diagnostic workup by a psychologist showed significant attention-deficit with normal intellectual functioning. Signed informed consent was obtained from the patient’s parents.

### Therapy

The therapy was divided into three phases. During the first phase, pyridoxine was given at the dose of 500 mg daily with unsatisfactory clinical and biochemical effect. Plasma Orn concentration was measured at two-month intervals while the patient was fasting. In the second phase of therapy, the dose of pyridoxine was lowered to 2 x 100 mg daily and a low protein diet was implemented (at first, 1 g/kg/day of natural protein that was further gradually lowered to 0.6 g/kg/day). Lastly, during the third phase of treatment, the amino acid L-lysine was additionally administered. L-lysine was given in rising dosages from 6 to 12 g daily. The goal was to increase the excretion of Orn and Arg by urine in order to reduce plasma Orn concentration as low as possible.

### Sample collection

Whole blood was taken on two different anticoagulants, K_3_/K_2_-EDTA and lithium heparin, and in serum gel tubes (Greiner Bio-One GmbH®, Kremsmünster, Austria). For ammonia measurement, samples were collected and immediately put on ice after venepuction. Plasma and serum were removed from the cells by centrifugation at 2500xg for 10 minutes at room temperature (Thermo Scientific™, Osterode, Germany) within a few hours after blood collection and analysed immediately. Samples for amino acid analyses were taken in 5 mL tubes about four hours after meals. Urine samples were collected in 10 mL tubes without additives (Greiner Bio-One GmbH®, Kremsmünster, Austria).

### Biochemical and haematological analysis

Before starting therapy, complete blood count was performed using UniCel*®* DxH 800 Coulter® analyser (Beckman Coulter Inc., Brea, CA, USA). For routine biochemical analyses, Cobas c501/c311 (Roche Diagnostics®, Basel, Switzerland) was used.

### Quantitative amino acid analysis

Amino acids were measured with liquid chromatography coupled with electrospray tandem mass spectrometry LC-MS/MS (UPLC Nexera, Shimadzu, Canby, OR, USA; API 3200, Sciex, Framingham, MA, USA). The use of aTRAQ™ kit for physiological amino acid analysis (Sciex, Framingham, MA, USA) allows quantitation of free amino acids in plasma and urine. Cliquid Software in conjunction with Analyst^®^ Software (Sciex) applications was used to obtain data for the quantification of up to 45 amino acids. The instrument automatically calculates the concentration of amino acids in each sample. The values obtained from the urine sample must be divided by the creatinine concentration (mmol/mol creatinine).

**Figure 2:**
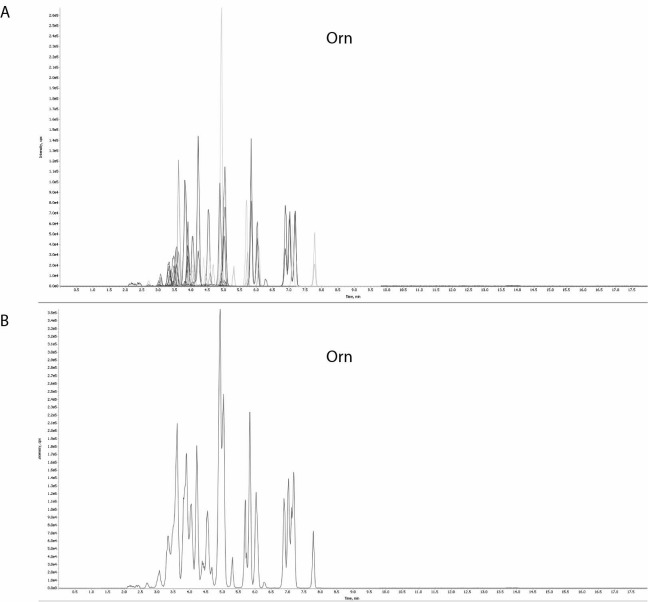
High intensity peak of ornithine (Orn) was found by comparing the patient’s plasma amino acid chromatogram (A) with a chromatogram of the internal standard (B).

Preparation of the samples was made according to manufacturer’s instructions using 40 μL of physiological fluids. They were introduced firstly into a liquid chromatographic system where they were separated on a chromatographic column (AAA C18 Column, 4.6x150 mm, Sciex, Framingham, MA, USA), and then into a mass spectrometer where they were ionized, fragmented and further separated. The ionized fragments were detected based on mass and ion charge ratio (m/z). Every ionized fragment generated was characterized by specific retention time. The individual amino acids were identified by characteristic fragments with specific mass transitions (m/z), while the quantitative analysis was performed by comparing the intensity of the patient amino acid ion with the corresponding internal standard (amino acid concentration) (Figure 2).

### Molecular genetic analysis

In order to confirm the diagnosis, a DNA sample of the patient was analysed in a collaborative laboratory (Division of Metabolism, University Children’s Hospital, Zürich, Switzerland). Genomic DNA was extracted from 1 mL whole blood using FlexiGene DNA Kit (Qiagen, Crawley, UK). Polymerase chain reaction (PCR) was carried out using the HotStar Taq DNA Polymerase kit (Qiagen, Hombrechtikon, Switzerland) on a Biometra T-Professional thermocycler (Biometra GmbH, Göttingen, Germany) with following cycling conditions: 1 cycle of 15 min at 95°C, followed by 35 cycles of 30 sec at 95°C, 20 sec at 63°C and 1 min at 72°C, and finishing with 7 min incubation at 72°C. The OAT coding exons including the flanking intronic regions were amplified by PCR using the following primers (Table 1). The size and quality of DNA fragments obtained was analysed by 2% agarose-gel-electrophoresis with EZ Vision One dye (VWR Amresco, Dietikon, Switzerland) in 5 x TBE buffer (45 mM Tris-borate, 1mM EDTA) at 100 V for 35 minutes. DNA fragments were visualized using the AlphaImager HP gel imaging system (Bio-Techne AG, Zug, Switzerland) under UV light wavelength of 365 nm. After agarose gel electrophoresis PCR products were Sanger sequenced according to standard protocols using the BigDye Terminator Cycle Sequencing kit v1.1 and an ABI 3130 Genetic Analyser (Applied Biosystems by Life Technologies Europe BV, Zug, Switzerland). Sequencing raw data were analyzed using the SeqPatient module of the SEQUENCE PILOT software (JSI Medical Systems, Ettenheim, Germany) using the following reference sequences: OAT Ensembl ENSG00000065154 and ENST00000368845.5. Nomenclature of the mutation follows the recommendations of the Human Genome Variation Society (*13*) and was checked using the online software Mutalyzer (*14*).

**Table 1:** Primer sequences

Exon Primer (5^,^----3^,^)	
**Forward**	**Reverse**
Exon 2 Fwd 5’-GTGATGGAGTCTTGCTCTGTTGC-3’	Exon 2 Rev 5’-CCATGTCTGCAATATACAC-3’,
Exon 3/4 Fwd 5’-GTGTGTTTTGAAGCTGGGCAG-3’,	Exon 3/4 Rev 5’-GAAGGCTGGTCTTGAACTCCAG-3’,
Exon 5 Fwd 5’-GAAGGTGCACTAAAGCAAGCC-3’,	Exon 5 Rev 5’-GAGAACAAGTCTGAAATCGTGGC-3’,
Exon 6 Fwd 5’-GGCAGTGAATTTGAAGTCAG-3’,	Exon 6 Rev 5’-GCTCTTAGAATGCCATCGC-3’,
Exon 7 Fwd 5’-GAGGGCACATCAGAATTACAC-3’,	Exon 7 Rev 5’-AGGCACACTCAATTCTTCAG-3’,
Exon 8 Fwd 5’-GAGTCCAGGAGGCAGAGATTG-3’,	Exon 8 Rev 5’-GAGGAAATCCAGTCTACTAGG-3’,
Exon 9 Fwd 5’-GCAAGACTCTGAGCTAGTGTATG-3’,	Exon 9 Rev 5’-CTGAGGCAGAGAATTGCTTG-3’,
Exon 10 Fwd 5’-CAATCTCTTGACCTCGTGATCC-3’,	Exon 10 Rev 5’-CGGCAGGTTCATAAACGTTG-3’.

## Results

Routine biochemical analysis and complete blood count results were within normal range (Table 2), except creatinine and ammonia that were below normal range (creatinine 23 µmol/L, reference interval 25 - 42 µmol/L; ammonia 20.9 µmol/L, reference interval 24.0 - 48.0 µmol/L).

**Table 2:** Patient’s biochemistry and complete blood count test results

**Analyte**	**Results**	**Unit**	**Reference interval**
Urea	3.0	mmol/L	1.8 - 6.0
Creatinine	23	µmol/L	25 - 42
Ammonia	20.9	μmol/L	24.0 - 48.0
K	4.7	mmol/L	3.6 - 5.0
Na	137	mmol/L	134 - 143
Cl	102	mmol/L	96 - 109
Ca	2.41	mmol/L	2.15 - 2.80
P	1.35	mmol/L	1.11 - 1.73
Mg	0.77	mmol/L	0.65 - 1.03
Albumin	47.2	g/L	35.0 - 52.0
Transferrin	2.38	g/L	2.00 - 3.60
RBC	4.58	x10^12^/L	4.00 - 5.00
Hb	127	g/L	109 - 138
Hct	0.373	L/L	0.320 - 0.404
MCV	78.3	fL	73.8 - 89.4
MCH	26.8	pg	24.3 - 29.2
MCHC	342	g/L	300 - 350
RDW	14.1	%	9.0 - 15.0
Rtc	9.1	/1000 RBC	4.0 - 19.0
Rtc	43	x10^9^/L	22 - 97
IRF	0.3		0.1 - 0.3
WBC	5.4	x10^9^/L	5.0 - 13.0
Eosinophils	3	%	0 - 6
Segmented Neutrophils	51	%	30 - 72
Lymphocytes	33	%	15 - 55
Monocytes	13	%	5 -13
Eosinophils	0.16	x10^9^/L	0.00 - 0.70
Segmented Neutrophils	2.75	x10^9^/L	1.40 - 8.00
Lymphocytes	1.78	x10^9^/L	1.40 - 5.00
Monocytes	0.70	x10^9^/L	0.22 - 1.51
PLT	198	x10^9^/L	150 - 450
MPV	8.9	fL	6.9 - 11.3
K - potassium. Na - sodium. Cl - chloride. Ca - calcium. P - inorganic phosphates. Mg - magnesium.RBC - red blood cell count. Hb - haemoglobin. Hct - haematocrit. RDW - red cell distribution width. MCV - mean corpuscular volume. MCH - mean corpuscular haemoglobin. MCHC - mean corpuscular haemoglobin concentration; Rtc - reticulocytes. IRF - immature reticulocytes fraction. WBC - white blood cell count. MPV - mean platelet volume. PLT - platelet count.			

Quantitative analysis of amino acids in plasma is necessary for patients suspected to have hyperornithinemia. Our patient had significantly increased initial concentrations of Orn (1039 μmol/L; reference interval 20 - 155 μmol/L) (Table 3).

**Table 3:** Long-term follow-up: plasma amino acids in the patient with gyrate atrophy

**Amino acids**	**Reference interval (µmol/L)**	**Before treatment (µmol/L)**	**After treatment (µmol/L)**		
			**First phase (vit.B_6_ 500 mg/day)**	**Second phase (vit.B_6_ 200 mg/day and low-protein diet)**	**Third phase (vit.B_6_ 200 mg/day, low-protein diet and L-lysine)**
Orn	20 - 155	1039	907	537	489
Ala	130 - 547	250	229	506	642
Arg	10 - 140	79	81	66	58
Asn	20 - 112	47	47	53	49
Asp	0 - 20	4	4	4	4
Cit	1 - 46	31	31	23	26
Gln	254 - 823	518	637	568	629
Glu	10 - 150	21	17	18	16
Gly	110 - 343	194	163	251	199
His	41 - 125	63	68	78	87
Hyp	0 - 45	26	28	21	15
Ile	22 - 107	61	68	43	39
Leu	49 - 216	110	125	75	75
Lys	50 - 284	74	72	201	218
Met	15 - 45	-	0	-	23
Phe	26 - 91	46	51	47	40
Pro	60 - 340	166	202	207	187
Ser	70 - 194	141	133	164	112
Tau	20 - 170	32	30	48	40
Thr	35 - 226	134	135	94	86
Tyr	25 - 115	58	65	51	36
Val	80 - 321	209	216	164	131
Orn - ornithine. Ala - alanine. Arg - arginine. Asn - asparagine. Asp - aspartic acid. Cit - citrulline. Gln - glutamine. Glu - glutamic acid. His - histidine. Hyp - hydroxyproline. Ile - isoleucine. Leu - leucine. Lys - lysine. Met - methionine. Phe - phenylalanine. Pro - proline. Ser - serine. Thr - threonine. Tyr - tyrosine. Val - valine.					

**Figure 3:**
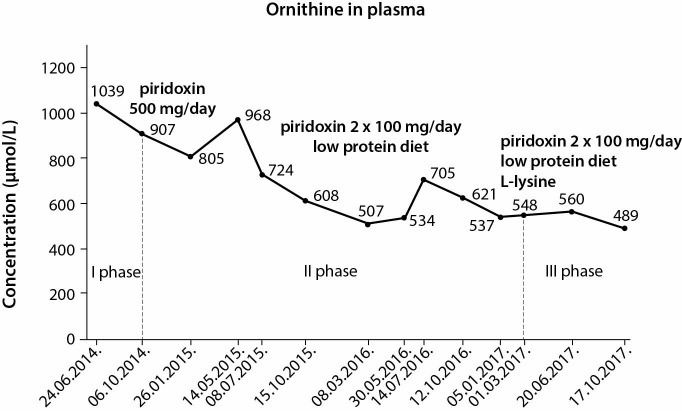
Monitoring of plasma amino acid concentration during dosage adjustment. Ornithine values significantly differed between the first and third period of treatment.

During the first phase of therapy, laboratory results of quantitative analysis of amino acids showed only a discrete decline in Orn concentration (13%). In the second phase of therapy, the decrease of plasma Orn was more significant (48%). The decrease of bilateral cystoid macular oedema was transient and the progression of peripheral atrophic lesions continued. During the third phase of treatment, adequate reduction of plasma Orn was achieved (53%) (Figure 3).

This resulted in increase of plasma lysine (Lys) concentration over the next two years from 74 to 218 μmol/L (reference interval 50 - 284 μmol/L) (Table 3).

Ornithine excretion in urine was measured at four-month intervals. Adding L-lysine supplement to a low protein diet resulted in increased excretion of Orn (153 mmol/mol creatinine, reference interval 0 - 7), Arg (36 mmol/mol creatinine, reference interval 0 - 7) and several other amino acids such as asparagine (Asn) (203 mmol/mol creatinine, reference interval 0 - 29), Lys (235 mmol/mol creatinine, reference interval 10 - 68), and citrulline (Cit) (9 mmol/mol creatinine, reference interval 0 - 5) in urine. In the third period during treatment with L-lysine, urinary excretion of Orn was highly increased. Administration of L-lysine increased renal excretion of Arg and Orn, which contributed to a decrease of plasma Orn.

The reduction of plasma Orn was associated with a positive change in the patient’s electroretinograms. During the third phase of therapy, the ophthalmologic fundus examination showed significant improvement (only discrete oedema of the macula was found).

Molecular genetic analysis revealed the homozygous mutation c.868_870delCTT in the exon 7 of the *OAT* gene resulting in the deletion of one amino acid p.(Leu290del). So far, this mutation has not been described in the Human Gene Mutation Database. The mutation (in frame deletion) was predicted to be pathogenic by *in silico* software: PROVEAN (Protein Variation Effect Analyser): score = - 10.25 (cut-off = - 2.5), prediction: deleterious.

## Discussion

Hyperornithinemia causes a permanent loss of retinal cells (atrophy) with the symptoms like myopia and progressive deterioration of peripheral and night vision, eventually resulting in blindness (*1*). Most patients with a diagnosis of GA have no symptoms other than vision loss, but in some cases neonatal hyperammonemia, intellectual disability, peripheral nerve problems and muscle weakness may occur (*15*). Our patient did not have any additional symptoms besides vision loss.

Besides high Orn concentration, low plasma concentrations of Lys, Glu, ammonia and creatinine are common characteristics of untreated GA (*1*). Our patient had mildly decreased concentrations of Lys and Glu (close to the lower reference limit). Plasma ammonia and creatinine were also reduced (below the reference interval). The reason for decreased concentration of ammonia and glutamate in untreated patients with GA is unknown.

An ordinary diet usually contains a high amount of protein, including amino acid Arg. However, Arg is an essential amino acid in GA patients and limitation of dietary Arg might result in reduction of plasma and urine Orn (*16*). The reduction of Arg intake had only transient benefit in our patient. The most benefit was achieved when L-lysine was added to the treatment. The dose of L-lysine was gradually increased until a plateau in ornithine plasma concentration was reached. In the study of Elpeleg *et al.* the administration of 10 - 15 g L-lysine daily for 40 - 55 days in three patients (ages 13 - 19) with gyrate atrophy decreased plasma Orn concentrations by 21 - 31%. A daily dose of 15 g was more effective than 10 g (*17*). It has been described in the literature that less than 5% of patients with GA had been responsive to vitamin B_6_ (*1*, *18*, *19*). A slight decrease in plasma Orn concentration was observed after supplementing vitamin B_6_ in our patient.

Recently, a new diagnostic test based on proline/citrulline ratio (Pro/Cit) in plasma and dried blood spot (DBS) in new-born screening laboratories was developed (*20*, *21*). Deficiency of OAT causes an increase in Pro/Cit ratio. Ornithine and Arg concentrations in plasma/DBS are often abnormal in neonates with OAT deficiency (Pro is increased, Cit is reduced) (*21*, *22*). This theory supports the use of the Pro/Cit ratio on tandem mass spectrometry with the best diagnostic sensitivity and accuracy. Proline/citrulline ratio should be considered as a differential diagnostic tool in new-born screening allowing differential diagnosis for all new-borns with hyperammonemia suspected of OAT deficiency (*22*). However, we have no possibility of verifying that information for our GA patient because our new-born screening laboratory in Croatia stores DBS for only five years. Current medical literature is lacking in data on early introduction of therapy in patients detected by new-born screening and patient’s follow-up. The reason for that could be relatively low disease incidence and probably low detection rate in less developed countries.

Patients with GA generally have normal intelligence (*23*). One of described patients had mild mental retardation, delayed language development, and speech defects (*24*). Our 10-year-old patient had normal intellectual functioning but with significant attention-deficit.

Hyperornithinemia is the main pathogenic factor in GA and our case proves that it can be treated by low protein diet, supplementation of vitamin B_6_ and L-lysine. To the best of our knowledge, this is the first report of GA in Croatian population. For the first time we introduce an approach of a stepwise L-lysine dosage adjustment guided by regular plasma Orn quantification, which resulted in the highest plasma ornithine reduction reported in the literature. The diagnosis was confirmed by molecular genetic analysis, with a new homozygous mutation detected in the *OAT* gene.

The purpose of this paper was to stress the importance of early diagnostics and treatment in GA. Laboratory professionals together with clinicians should consider introduction of new Pro/Cit ratio test in new-born screening programs since it could be valuable for the early diagnosis and treatment of GA patients, thus preventing occurrence of irreversible retinal changes.

It should be acknowledged that there are several limitations in this case report. The follow-up period is relatively short to show long-term outcome of the treatment on patient’s vision, although ophtalmological follow-up did show promising results. This is a follow-up study of only one patient. A larger number of treated patients might give us better insight into the efficacy of this treatment approach. As the disease is very rare (approximately 150 individuals with GA have been identified so far) multicentre collaboration would be needed to achieve that (*18*). For improvement of this study it should be useful to screen more subjects. The introduction of new-born screening for OAT deficiency should be helpful in early detection and early treatment introduction. Our patient is homozygous for a previously undescribed mutation expected to be pathogenic due to clear clinical phenotype (pathognomonic ocular changes), quantitative amino acid analysis and *in silico* analysis. For final proof of pathogenicity, further functional studies are needed to validate our results. As parental DNA was unavailable, we examined only our patient; consequently, it is not certain if this mutation is *de novo* mutation. Finally, Sanger sequencing by capillary electrophoresis was used. It is currently the most widely used routine molecular analysis and is adequate for the majority of clinical applications involving the analysis of single genes. The method is rapid, robust and known as a gold standard for clinical research sequencing with 99.9% accuracy, so it was not necessary to confirm this mutation with a second different molecular analysis (*25*).

In conclusion, if therapy for GA is introduced at an early age, along with long-term monitoring of plasma Orn concentrations, the progression of retinal function loss may be notably delayed. Combined treatment with low protein diet, L-lysine and pyridoxine supplementation in our patient resulted in plasma Orn decrease to 53% of the initial concentration and a significant improvement of ocular changes. Although longer follow-up is needed to estimate true benefits of this treatment, we find results presented in this paper very encouraging.

## References

[1] Valle D, Simell O. The hyperornithinemias. In: Scriver CR, Beaudet AL, Sly WS, eds. The metabolic and molecular bases of inherited disease. New York: McGraw-Hill; 2001. p.1875–95.

[2] Takki K Simell O Genetic aspects in gyrate atrophy of the choroid and retina with hyperornithinaemia. Br J Ophthalmol. 1974;58:907–16. 10.1136/bjo.58.11.907 4457103 PMC1215056

[3] Kaiser-Kupfer MI Ludwig IH de Monasterio FM Valle D Krieger I Gyrate atrophy of the choroid and retina. Early findings. Ophthalmology. 1985;92:394–401. 10.1016/S0161-6420(85)34022-8 3991128

[4] Ohkubo Y Ueta A Ito T Sumi S Yamada M Ozawa K Vitamin B6-responsive ornithine aminotransferase deficiency with a novel mutation G237D. Tohoku J Exp Med. 2005;205:335–42. 10.1620/tjem.205.335 15750329

[5] Mitchell GA Looney JE Brody LC Steel G Suchane M Engelhardt JF Human ornithine-delta-aminotransferase. cDNA cloning and analysis of the structural gene. J Biol Chem. 1988;263:14288–95. 3170546

[6] Barrett DJ Bateman JB Sparkes RS Mohandas T Klisak I Inana G Chromosomal localization of human ornithine aminotransferase gene sequences to 10q26 and Xp11. 2. Invest Ophthalmol Vis Sci. 1987;28:1037–42. 3596985

[7] Ramesh V Eddy R Bruns GA Shih VE Shows TB Gusella JF Localization of the ornithine aminotransferase gene and related sequences on two human chromosomes. Hum Genet. 1987;76:121–6. 10.1007/BF00284906 2886418

[8] Hayashi H Katunuma N Chiku K Endo Y Natori Y Cell-free synthesis of ornithine aminotransferase of rat liver. J Biochem. 1981;90:1229–32. 10.1093/oxfordjournals.jbchem.a133577 7309717

[9] ClinVar. Available at: https://www.ncbi.nlm.nih.gov/clinvar. Accessed April 8th 2018.

[10] Takki KK Milton RC The natural history of gyrate atrophy of the choroid and retina. Ophthalmology. 1981;88:292–301. 10.1016/S0161-6420(81)35031-3 7254775

[11] Kaiser-Kupfer M Kuwabara T Uga S Takki K Valle D Cataracts in gyrate atrophy: clinical and morphologic studies. Invest Ophthalmol Vis Sci. 1983;24:432–6. 6832916

[12] Kaiser-Kupfer MI de Monasterio FM Valle D Walser M Brusilow S Gyrate atrophy of the choroid and retina: improved visual function following reduction of plasma ornithine by diet. Science. 1980;210:1128–31. 10.1126/science.7444439 7444439

[13] Nomenclature by J-T.Den Dunnen. Available at: http://varnomen.hgvs.org/ recommendations/DNA/. Accessed April 8th 2018.

[14] Name Checker Mutalyzer 2.0.26. Available at: https://mutalyzer.nl/name-checker. Released on July 19th 2017.

[15] Sipilä I Simell O Rapola J Sainio K Tuuteri L Gyrate atrophy of the choroid and retina with hyperornithinemia: tubular aggregates and type 2 fiber atrophy in muscle. Neurology. 1979;29:996–1005. 10.1212/WNL.29.7.996 572946

[16] Valle D Walser M Brusilow SW Gyrate atrophy of the choroid and retina: amino acid metabolism and correction of hyperornithinemia with an arginine-deficient diet. J Clin Invest. 1980;65:371–8. 10.1172/JCI109680 7356686 PMC371375

[17] Elpeleg N Korman SH Sustained oral lysine supplementation in ornithine delta-aminotransferase deficiency. J Inherit Metab Dis. 2001;24:423–4. 10.1023/A:1010545811361 11486915

[18] Wirtz MK Kennaway NG Weleber RG Heterogeneity and complementation analysis of fibroblasts from vitamin B6 responsive and non-responsive patients with gyrate atrophy of the choroid and retina. J Inherit Metab Dis. 1985;8:71–4. 10.1007/BF01801668 3939534

[19] Javadzadeh A Gharabaghi D Gyrate atrophy of the choroid and retina with hyper-ornithinemia responsive to vitamin B6: a case report. J Med Case Rep. 2007;1:27. 10.1186/1752-1947-1-27 17565677 PMC1904451

[20] de Sain-van Der Velden MG Rinaldo P Elvers B Henderson M Walte JH Prinsen BHCMT The proline/citrulline ratio as a biomarker for OAT deficiency in early infancy. JIMD Rep. 2012;6:95–9. 10.1007/8904_2011_122 23430945 PMC3565682

[21] Cleary MA Dorland L De Koning TJ Poll-The BT Duran M Mandell R Ornithine aminotransferase deficiency: Diagnostic difficulties in neonatal presentation. J Inherit Metab Dis. 2005;28:673–9. 10.1007/s10545-005-0074-1 16151897

[22] Ginguay A Cynober L Curis E Nicolis I Ornithine aminotransferase, an important glutamate-metabolizing enzyme at the crossroads of multiple metabolic pathways. Biology (Basel). 2017;6:18. 10.3390/biology6010018 28272331 PMC5372011

[23] Peltola KE Jaaskelainen S Heinonen OJ Falck B Nanto-Salonen K Heinanen K Peripheral nervous system in gyrate atrophy of the choroid and retina with hyperornithinemia. Neurology. 2002;59:735–40. 10.1212/WNL.59.5.735 12221166

[24] Stoppoloni G Prisco F Santinelli R Tolone C Hyperornithinemia and gyrate atrophy of choroid and retina: report of a case. Helv Paediatr Acta. 1978;33:429–33. 711502

[25] Anderson MW Schrijver I Next generation DNA sequencing and the future of genomic medicine. Genes (Basel). 2010;1:38–69. 10.3390/genes1010038 24710010 PMC3960862

